# Development of a questionnaire to measure teachers’ student-centred perspectives based on the Onion Model

**DOI:** 10.1186/s12909-022-03547-9

**Published:** 2022-06-27

**Authors:** Lukas Daniel Leatemia, Jeroen J. G. van Merrienboer, Astrid Pratidina Susilo

**Affiliations:** 1 Department of Medical Education, Faculty of Medicine, Mulawarman University, Samarinda, East Kalimantan Indonesia; 2 Department of Educational Development and Research, Faculty of Health Medicine and Life Sciences, Maastricht University, Maastricht, the Netherlands; 3 Department of Medical Education and Bioethics, Faculty of Medicine, Universitas Surabaya, Surabaya, East Java Indonesia

**Keywords:** Teachers’ student-centred perspectives, Problem-based learning, Onion Model, Internal validation, External validation

## Abstract

**Background:**

Teachers with a teacher-centred perspective have difficulties applying student-centred approaches in Problem Based Learning (PBL) because they are inclined to show teacher-centred behaviours. The six aspects explained in Korthagen’s Onion Model (environment, behaviour, competencies, beliefs, identity, and mission) are assumed to contribute to teachers’ perspectives, showing that both the environment and personal characteristics influence behaviours. For teachers to function properly in PBL, those six aspects should reflect a student-centred perspective. Previous instruments to measure teaching perspectives focused on only a few of these relevant aspects. Therefore, we developed the Student-Centred Perspective of Teachers (SCPT) questionnaire with subscales for each aspect in the Onion Model. This study aimed to provide evidence for its internal and external validity.

**Methods:**

The SCPT was distributed in a survey to 795 teachers from 20 medical schools. For the internal validation, Confirmatory Factor Analysis was performed to analyse theoretical fit model validation, convergent validation, and discriminant validation. For the external validation, teachers’ perspective scores were compared among three groups of amount of PBL training using Analysis of Variance (ANOVA) and post-hoc Least Significant Difference (LSD) tests. The *p*-value for all tests was set at .05.

**Results:**

A total of 543 out of 795 teachers (68.3%) participated. Confirmatory Factor Analysis showed the evidence of the SCPT’s internal validation with acceptable fit for the six subscales measured by 19 items and the following Composite Reliability scores: environment (.72), behaviour (.74), competencies (.63), beliefs (.55), identity (.76), and mission (.60). All items’ factors loadings reached a good standard (.5 or greater). Only the environment subscale had the Average Variance Extracted (AVE) score higher than .5 and the Maximum Shared Variance score lower than the AVE score. ANOVA and Post-hoc LSD tests showed that teachers who participated in more PBL training showed significantly higher student-centred perspectives, providing evidence for external validity.

**Conclusion:**

The SCPT is a reliable and valid instrument to measure teaching perspectives. Identifying aspects that do not represent the adoption of a student-centred perspective may provide valuable input for faculty development in the context of PBL.

## Background

Many medical faculties from all over the world have implemented problem-based learning (PBL). In the implementation, all educational program components, including teachers, should be consistent with student-centred teaching and learning behaviours [1]. However, studies showed that many teachers tend to use teacher-centred approaches and do not properly facilitate students’ learning in PBL. They have difficulties moving away from the hierarchical student–teacher relationship [2–5].

One reason for teachers not showing desired behaviours in PBL is related to their teaching perspectives. Teachers with a teacher-centred perspective are assumed to have difficulties showing student-centred approaches. Therefore, institutions need to identify teachers’ teaching perspectives. Only then the institutions can remove the obstacles to the effective implementation of PBL, for example, through faculty development [6]. An appropriate instrument to measure the teaching perspectives is necessary with attention to all aspects that influence student-centred versus teacher-centred behaviour. This reported study aims to validate an instrument that will help the institutions recognise teachers’ barriers in showing student-centred behaviours.

To perform student-centred behaviour in PBL is challenging for teachers with a teacher-centred perspective because newly required behaviour is not in line with their convictions [7]. Pratt et al. [8] pointed out that a teaching perspective is a teacher's view about teaching in which the interrelation of beliefs and intentions provides direction and justification for actual behaviour. It means that teachers with a student-centred perspective tend to show student-centred behaviour. They focus on student development and student-centred learning. Conversely, teachers who have a teacher-centred perspective tend to take a teacher-centred approach. They focus on their task to transmit knowledge based on the syllabus or textbook without acknowledging the student's experiences and understanding [9].

**Fig. 1:**
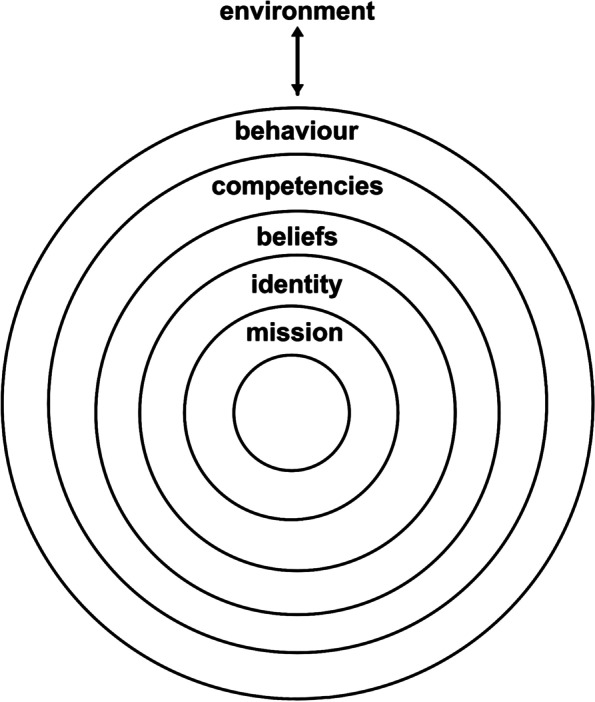
The Onion Model [10]

Korthagen [10] pointed out six aspects (environment, behaviour, competencies, beliefs, identity, and mission) that contribute to teachers’ perspectives, showing that both the environment and personal characteristics influence behaviours. These six aspects, including the behaviour itself, are structured as the six layers resembling a sliced onion, in the so-called Onion Model (Fig. 1). The Onion Model illustrates that the inner levels influence the outer levels and vice versa (from outside to inside).

The teaching perspectives relating to the *environment* refer to the external side that the teacher encounters in his/her institution, such as support from a leader and departmental peers, the number of students in a small PBL group, teaching and learning facilities, institutional rewards for teachers, and opportunities for personal development [11–13]. *Behaviour* refers to the teaching activities in small PBL group sessions, such as the stimulation of constructive/active learning, self-directed learning, contextual learning, and the collaborative learning of students [14]. *Competencies* refer to the knowledge, skills, and attitudes of teachers for stimulating and asking questions, providing information, observing and analysing, and providing feedback [15]. *Beliefs* refer to the teaching and learning values of teachers with regard to student learning in small group discussions [16]. *Identity* refers to how the teacher defines or sees his/her professional identity as didactic and pedagogical expertise [17, 18]. *Mission*, finally, refers to a teacher's personal and professional aims and aspirations, such as care for the whole person, temperance, and humility [19].

Paying attention to these six aspects in measuring the teaching perspectives of teachers might help educational institutions identify obstacles that teachers face and recognise faculty development needs. To function properly in PBL, teachers' teaching perspectives, as measured by those six aspects, should be in a student-centred direction. When one or more of the aspects show a teacher-centred direction, they indicate an obstacle to performing student-centred behaviours. This finding will help educational institutions recognise the needs of individual teachers and, especially, how faculty development might help address those needs [6].

Instruments that use the Onion Model as their theoretical framework for measuring teachers' teaching perspectives do not yet exist. Previous instruments to measure teachers’ teaching perspectives, such as the Learning Inventory [20], the Approaches to Teaching Inventory (ATI) [9], the Teaching Perspectives Inventory (TPI) [21], and the Conceptions of Learning and Teaching (COLT) [16] focused on only a few of these relevant aspects, such as student learning, teacher learning, teaching approach, or teachers' uncertainty. That is problematic because the sole focus on teaching and learning processes might lead institutions to implement faculty development programs that neglect the required transformation of teachers’ beliefs, identity, and mission [6, 22].

A new instrument named Student-Centred Perspectives on Teaching (SCPT) is proposed based on the Onion Model's six levels to measure teachers' teaching perspectives. A sound instrument should have acceptable internal and external validity. Internal validation of the questionnaire will be conducted to confirm that the SCPT can quantitatively measure teachers' teaching perspectives based on the six aspects, the convergence or the sharing proportion of the items within a subscale, and the distinction of a subscale from other subscales. In addition, external validation will be conducted to provide evidence that the SCPT can distinguish teachers' teaching perspectives based on the six aspects according to the amount of PBL training they have been involved in. Therefore, the research question is: what is the evidence to support the internal and external validity of the SCPT?

## Methods

### Setting

This study took place in 2020 and data were collected in 20 medical schools spread over Indonesia from May to July. These medical schools were randomly selected from 90 medical schools in Indonesia, being representative of medical schools in six areas of the Indonesian Medical Education Association (IMEA): Area 1 (Sumatera), Area 2 (Jakarta), Area 3 (West Java), Area 4 (Central Java, Jogjakarta and Kalimantan), Area 5 (East Java, Bali and West and East Nusa Tenggara), and Area 6 (Sulawesi, Maluku and Papua). Twenty selected schools were considered adequate to obtain the minimum number of participants.

All the selected medical schools have implemented a PBL curriculum due to a regulation introduced by the Indonesian government [23]. Most have implemented a hybrid curriculum with a mix of traditional lectures and PBL sessions. The number of lectures varies for each school while most of them conduct one PBL session per week throughout the academic years. One session of PBL consists of two small group meetings and self-study for two or three days in between. The schools use procedures adopted from the Seven-Jump of the PBL process as developed at Maastricht University. In this procedure, teachers facilitate students’ discussion to learn from a problem (paper-based scenario), define learning objectives, and then refine acquired knowledge [5].

At the beginning of PBL implementation, all the medical schools conducted formal PBL training in the traditional format (i.e., seminars and workshops) to increase teachers’ competencies to work as tutors. The training was typically offered for one to two days, 6–7 h per day, and training activities vary and include lectures, practical work, and discussion. The training was a prerequisite for all their teachers before they worked as a tutor for the first time. However, there were some barriers for teachers to participate. Some teachers could not attend the whole training because they had other clinical and teaching tasks. In addition, after several years of PBL implementation, several institutions do not routinely conduct PBL training. Consequently, there is a possibility for new teachers to work as tutors without having formal PBL training experience.

### Participants

The participants were all teachers from the selected schools that fulfilled the required criteria. The professional requirements for selected participants were: (a) full-time teachers, (b) actively involved in tutoring PBL sessions, and (c) satisfy ‘a’ and ‘b’ for more than one year. The selected schools sent the participants’ data (i.e., names and contact numbers and email addresses) to the author when they agreed to join this study. We included all these teachers as the target participants. The minimal number of participants was set at 10–15 teachers per measured variable, that is, 440 participants [24].

### The SCPT questionnaire

The SCPT questionnaire used for internal and external validation originally consisted of 44 items (SCPT-44). The questionnaire contained six subscales: environment, behaviour, competency, belief, identity, and mission. The 5-point Likert scale items were constructed based on the Onion Model, with answer options ranging from 1 = strongly disagree to 5 = strongly agree. The higher the score, the more student-centred the teachers’ perspective is. The SCPT-44 was developed by the first author and six local experts with master’s or doctoral degrees in medical education checked the content and clarity of items. In addition to the 44 items, the questionnaire also included several questions regarding the participants’ personal characteristics (age, gender, academic discipline and educational background) and the amount of PBL training the participant had undergone.

### The validation process

**Fig. 2:**
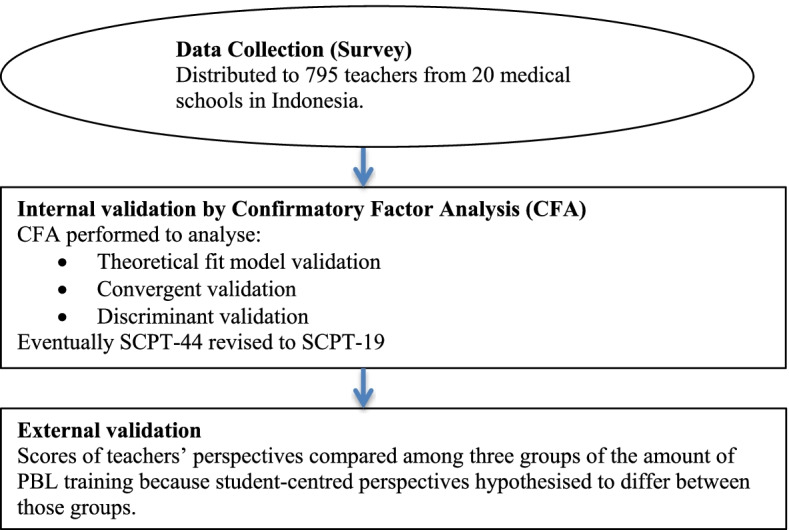
The questionnaire development process with internal and external validation. SCPT = Student-Centred Perspective of Teachers questionnaire, SCPT-44 = SCPT consisted of 44 items, SCPT-19 = SCPT consisted of 19 items

The process to determine internal and external validity and, if necessary, to revise the SCPT-44 is presented in Fig. 2. The SCPT-44 was distributed in a survey to 795 teachers from 20 medical schools. The SCPT-44 was distributed using the Qualtrics^XM^ application. Reminders were sent on day 3 and day 10 after the questionnaire was sent to participants.

The data from the survey were analysed using CFA in Amos version 25. The theoretical fit model validation was conducted based on the model fit indices: chi-square divided by degrees of freedom (*x*^*2*^*/df*), *p*-value, the goodness-of-fit index (GFI), the adjusted goodness-of-fit (AGFI), Root Mean Square Error of Approximation (RMSEA), and Comparative Fit Index (CFI). Indicators of a good model fit were *x*^*2*^*/df* < 3 with a non-significant *p*-value, GFI ≥ 0.95, AGFI ≥ 0.90, RMSEA value < 0.08, and CFI value ≥ 0.90 [25, 26].

Based on the CFA results, the convergence of the items and the discriminancy of the subscales were assessed. Both were conducted simultaneously. Factor loadings, composite reliability (CR), and Average Variance Extracted (AVE) were calculated to indicate whether the items in each subscale converged. Indicators of convergent validity are a factor loading of 0.5 or higher, CR of 0.7 or higher (or between 0.6 and 0.7 is acceptable), and AVE of 0.5 or higher. Maximum Shared Variance (MSV) was calculated to evaluate the separation of a subscale from other subscales. The MSV score of each subscale was compared with the AVE score. The subscales are considered discriminant if the AVE value is greater than the MSV value [26].

The external validation was conducted with a revised version of the questionnaire (SCPT-19), by dividing the data from the survey into three groups based on the amount of PBL training: (1) participants who did not have any experience in PBL training (non-training group), (2) participants who had undertaken PBL training programs one to two times (moderate-training group), and participants who had joined PBL training programs three or more times (high-training group). The normality of the data was assessed with the Kolmogorov–Smirnov, Skewness, and Kurtosis tests. Means and standard deviations of the teachers’ perspective scores were calculated and compared using ANOVA and post-hoc Least Significant Difference (LSD) tests. The *p*-value for all tests was set at 0.05 [24].

### Ethical consideration

Informed consent was obtained from all participants. All identities were kept confidential and not revealed in the study reports and any related publications. Ethical clearance was obtained from the Ethical Committee of Abdul Wahab Sjahranie Hospital, East Kalimantan, Indonesia, with the approval number 179/KEPK-AWS/I/2020.

## Results

A total of 543 out of 795 invited teachers (68.3%) participated in this survey. During the preliminary analysis, three participants were eliminated as they had missing values for more than five items. The data from 20 participants with fewer than five missing values were imputed with the mean score of the items in the subscale. At the end of the data screening, 540 out of 543 participants (99.4%) remained in the sample. Twenty-one participants (3.9%) had no PBL training experience, 314 participants (58.1%) had undergone PBL training programs one or two times, and 205 participants (38.0%) had joined PBL training programs three or more times. Eighty-one per cent of participants in the non-training group had less than 5 years of experience as a teacher. The participants’ characteristics are presented in Table 1.

**Table 1:** Participants’ characteristics

	**Amount of PBL Training**		
**Variable**	**None (*n* = 21)**	**Moderate (*n* = 314)**	**High (*n* = 205)**
Age (years, mean, SD)	35.9 (7.3)	39.4 (8.1)	44.2 (8.9)
Gender (n, %)			
Male	7 (33.3)	103 (32.8)	63 (30.7)
Female	14 (66.7)	211 (67.2)	142 (69.3)
Disciplines (*n*, %)			
Clinicians	13 (61.9)	115 (36.6)	62 (30.2)
Basic science	3 (14.3)	120 (38.2)	91 (44.4)
Others	5 (23.8)	79 (25.2)	52 (25.4)
Educational background *(n*, %)			
Bachelor degree	4 (19.0)	45 (14.3)	5 (2.4)
Master degree	16 (76.2)	233 (74.2)	144 (70.3)
Doctoral degree	1 (4.8)	36 (11.5)	56 (27.3)

*Note*: None = participants had no experience in PBL training programs; Moderate = participants followed PBL training programs one to two times; High = participants followed PBL training programs three or more times

After the preliminary analysis, the data of the 540 participants were included in the CFA for the internal validation (the theoretical fit model, convergent validation, and discriminant validation), and the external validation.

### Internal validation

In the initial CFA, the theoretical fit model validation showed that the 6-factor model had a poor fit except for the RMSEA values (see Table 2). Therefore, the model was revised iteratively to obtain a better fit.

**Table 2:** The fit indices of the models at the initial assessment

**Model**	***x***^***2***^	***df***	***x***^***2***^***/df***	***P***	**GFI**	**AGFI**	**CFI**	**RMSEA**
The 6-factor model	2321.18	887	2.62	.000	0.80	0.78	0.80	0.06
The revised 6-factor model	258.61	136	1.71	.000	0.95	0.93	0.96	0.04

**Fig. 3:**
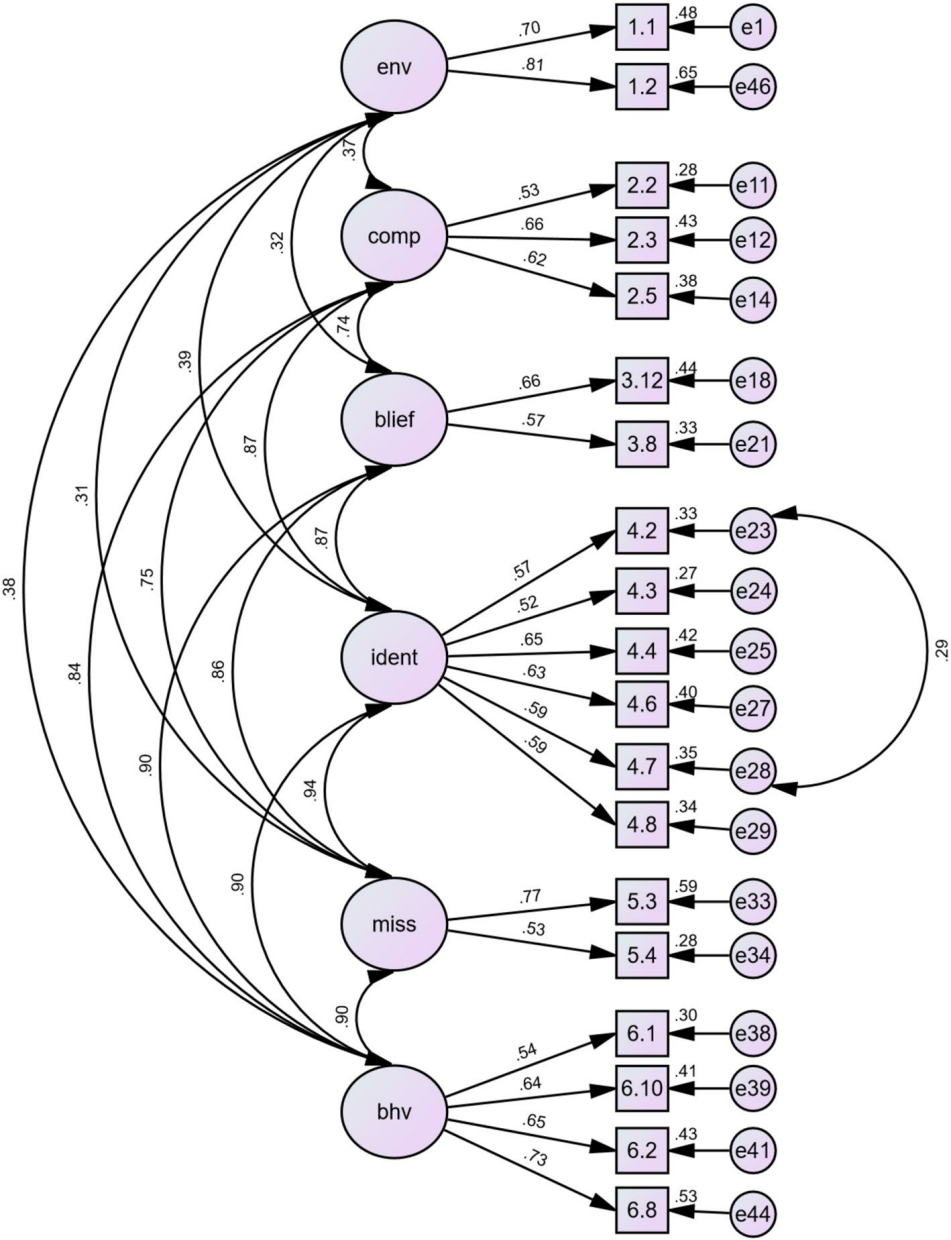
The structure model of the SCPT-19. Env = environment, comp = competency, blief = belief, ident = identity, miss = mission, bhv = behavior

The first step in the revision was the assessment of factor loadings. In this step, 16 items were deleted because their factor loadings were lower than 0.5. However, after this step, the model still did not have an acceptable fit based on the fit indices scores. The second step was the assessment of MI and SR. The iterative assessments of MI and SR values were conducted for the best improvement of the model. Three cross paths were added based on the MI evaluation, and nine items were removed based on the SR scores. The items with SR values higher than 2.5 were prioritised for removal. After the deletion of 25 items, the revised model was deemed acceptable, as indicated by the fit indices (second row in Table 2). The six subscales with 19 items (SCPT-19) were sustained. The subscales and the 19 items with their factor loadings are listed in Table 3, while the revised model structure is shown in Fig. 3.

**Table 3:** Results of item-factor loading for the SCPT-19

**Subscale**	**Items**	**Factor loading**
Environment	• My institution facilitates discussion with all lecturers to discuss PBL (views/concepts, small group discussion processes, etc.) routinely	.695
	• My institution evaluates the implementation of the PBL curriculum periodically through a specific unit/agency	.806
Behaviour	• I encourage my students to make summaries using their own words	.543
	• I encourage my students to develop the correlation of concepts discussed in tutorial groups	.653
	• I encourage students to apply their knowledge to the issues discussed	.731
	• I encourage my students to link their learning goals with their prior knowledge they have	.643
Competency	• I have the ability to stimulate student discussion using formal and informal communication	.527
	• I am able to ask open-ended questions to give students a better understanding of the task	.657
	• I am able to provide practical examples using language that is easily understood in tutorial discussion	.616
Belief	• Learning in a small group discussion encourages students to learn	.571
	• Group discussion of a topic will help students learn about how to get a deep understanding from various points of view	.665
Identity	• I am happy to provide assistance in solving learning problems faced by students	.573
	• I consider the needs of students when facilitating the small group discussions	.519
	• It is important for me to help students apply what they have learned in their daily lives	.648
	• It is important for me to have a caring personal relationship with students in the tutorial process	.630
	• I feel satisfied if I can help students when they experience problems	.592
	• The most important thing for me is creating a classroom atmosphere that makes students feel valued	.586
Mission	• I am open to new ideas and experiences	.767
	• In discussion with my students, I do not feel disturbed by opinions that differ from mine	.528

The final version of the scale adequately covers the scope of the Onion Model. Table 3 shows that the SCPT-19 consists of two items from the environment, four items from the behaviour, three items from the competencies, two items from the beliefs, six items from the identity, and two items from the mission. The two items of the environment refer to the leadership (i.e., support from a leader) while the four items of the behaviour refer to the stimulation of constructive/active learning and contextual learning. The three items of the competencies refer to the skills of stimulating and asking questions and providing information while the two items of the beliefs refer to teachers’ values on the student-centredness of student learning. The six items of the identity refer to the didactical and pedagogical experts while the two items of the mission refer to the care for the whole person.

The CFA continued for the convergent and discriminant validation of the SCPT-19. Factor loadings, CR, and AVE scores are indicators of the convergent validation. All factors loadings reached a good standard (0.5 or greater). Only the belief subscale had a CR score lower than 0.6 (0.55), and only the environment subscale had an AVE score higher than 0.5. The environment subscale had a MSV score lower than the AVE score in the discriminant validation, while those of other subscales had higher MSV than AVE scores (Table 4).

**Table 4:** CR, AVE, and MSV of the SCPT-19

**Factor**	**Number of items**	**CR**	**AVE**	**MSV**
Environment	2	.72	.57	.15
Behavior	4	.74	.42	.82
Competency	3	.63	.36	.75
Belief	2	.55	.38	.81
Identity	6	.76	.35	.88
Mission	2	.60	.43	.88

*Note*: *CR* Composite reliability, *AVE* Average Variance Extracted, *MSV* Maximum Shared Variance

### External validation

The mean scores and standard deviation of the six subscales in each amount-of-training group (no training, moderate, high) are presented in Table 5. The total mean scores of the teachers' perspectives in all the subscales were higher than the neutral score of 3. ANOVA showed that the amount of training had a significant effect on all subscales. Post-hoc LSD tests showed that the subscale scores of the high-training group were higher than the corresponding scores of the moderate-training group. Meanwhile, the scores of the high-training group were higher than the scores of the no-training group for the environment, behaviour, and competency subscales.

**Table 5:** Teachers’ perspectives scores in the six subscales among the three PBL training groups

**Subscale**	**Mean (SD) Total (*N* = 540)**	**Mean (SD) Amount of PBL Training**			***F*-value**	***p*-value**	***LSD (Significant difference)***
		**None (*n* = 21)**	**Moderate (*n* = 314)**	**High (*n* = 205)**			
Environment	3.85 (0.77)	3.38 (0.69)	3.74 (0.81)	4.06 (0.68)	15.03	.000	None vs moderate, none vs high, moderate vs high
Behavior	4.30 (0.48)	4.08 (0.72)	4.25 (0.41)	4.39 (0.40)	9.46	.000	None vs high, moderate vs high
Competency	4.07 (0.45)	3.82 (0.48)	4.04 (0.43)	4.15 (0.47)	7.08	.001	None vs moderate, none vs high, moderate vs high
Belief	4.38 (0.46)	4.55 (0.42)	4.34 (0.44)	4.43 (0.49)	3.48	.031	Moderate vs high
Identity	4.24 (0.43)	4.16 (0.37)	4.19 (0.43)	4.31 (0.43)	5.59	.004	Moderate vs high
Mission	4.30 (0.48)	4.14 (0.69)	4.27 (0.46)	4.35 (0.49)	3.12	.045	Moderate vs high

## Discussion

This study provides evidence for the internal and external validity of the SCPT based on data from 20 medical schools in Indonesia. The internal validation has confirmed that the SCPT with 19 items can measure the student-centred perspective, based on the six levels of the Onion Model. The external validation showed that the SCPT can distinguish the teachers' perspectives on these aspects by the different amounts of PBL training undergone by teachers. The more PBL training the teachers had participated in, the higher their scores for student-centred perspective on all of the six aspects.

The findings support the theoretical framework of the Onion Model in terms of its structure and the interrelation among the aspects. The environment that reflects the institutions' roles for teachers’ work in PBL is the external layer of the Onion Model. The other aspects (behaviour, competencies, beliefs, identity, and mission) are the internal layers, reflecting the personal characteristics of a teacher. The aspects in the internal layers are interrelated to each other, meaning that all those layers influence each other and ideally should have a similar direction. This interrelation of the aspects is important to identify the obstacles of teachers for showing the desired behaviour. When one or more aspects are not in the desired direction, the obstacles of teachers will be identified [10].

Paying attention to the six aspects when measuring teaching perspectives provides an opportunity for targeted teacher training. All of the aspects are of fundamental importance to faculty development [10]. However, many institutions often ignore to intervene in teachers’ beliefs, professional identity, and mission in faculty development [10, 12, 22]. Paying attention to the six aspects will support institutions to intervene in teachers' obstacles in a more holistic approach to faculty development [10, 22, 27].

The SCPT can be implemented for identifying faculty development needs. The external validation of the SCPT and the interrelation of the aspects help to understand the relationship between the teachers' teaching perspectives and faculty development. In the external validation, we found that PBL training might help change teachers’ teaching perspectives. The validation provides evidence that the SCPT can measure the change of teachers’ teaching behaviour after joining one or more training programs or other faculty development activities. In addition, there is a possibility for the SCPT to identify teachers' obstacles for faculty development needs by measuring the similarity of the student-centred direction and the relationship among all of the aspects. For this aim, further study is necessary. Recognising these obstacles will provide valuable input for faculty development of teachers [6].

There are several strengths of this study. First, this study used several strategies to construct the SCPT, such as the involvement of six local experts in medical education to develop the items, the internal validation with three analysis methods of CFA (theoretical fit model, convergent, and discriminant validation), as well as the external validation, resulting in a valid instrument to measure teachers’ teaching perspectives in medical schools with PBL curricula. Second, although the participation rate is 68.3%, the number of participants has reached a good sample size. The suggested sample size is at least 10–15 participants per variable [24]. Third, the SCPT may help teachers and institutions to see all the aspects of their professional lives that contribute to the teachers' teaching perspectives in not more than five minutes.

This study has several limitations. First, many items had to be removed to obtain an acceptable fit of the six-factor model. However, to guarantee that only good items are retained in the instrument, we kept the cut-off values of factor loadings 0.5 and the SR 2.5 [25, 26]. Second, there is a possibility for the participants to give socially desirable answers to the items in the questionnaire. However, authors have attempted to minimise this limitation by using reversed statements for several items [24]. Third, the kind and duration of PBL training in each institution were not considered in this study. For this aim, a further study to identify the teacher's student-centred perspective in different kinds and duration of the training programs is necessary [22].

## Conclusion

The SCPT is a reliable and valid instrument to measure the student-centred perspectives on the six aspects of teachers’ professional lives (environment, behaviour, competency, belief, identity, and mission) and to distinguish the teachers' perspectives on these aspects by the different amounts of PBL training undergone by teachers. Using the SCPT may help teachers and institutions recognise individual teachers' needs and then plan suitable faculty development activities tailored to their needs.

## Data Availability

The datasets used during the current study are available from the corresponding author on reasonable request.

## Abbreviations

PBL: Problem Based Learning
SCPT: Student-Centred Perspective of Teachers
CFA: Confirmatory Factor Analysis
GFI: Goodness-of-Fit Index
AGFI: Adjusted Goodness-of-Fit Index
RMSEA: Root Mean Square Error of Approximation
CFI: Comparative Fit Index
CR: Composite Reliability
AVE: Average Variance Extracted
MSV: Maximum Shared Variance
LSD: Least Significant Difference
ANOVA: Analysis of Variance
